# Nematode-secreted peptides and host factor mimicry

**DOI:** 10.1093/jxb/ery144

**Published:** 2018-05-25

**Authors:** Yanfeng Hu, Tarek Hewezi

**Affiliations:** 1 Department of Plant Sciences, University of Tennessee, Knoxville, TN, USA; 2 Key Laboratory of Mollisols Agroecology, Northeast Institute of Geography and Agroecology, Chinese Academy of Sciences, Harbin, China

**Keywords:** Abscission, IDA, ligand peptide, Meloidogyne incognita, MiIDL1, molecular mimicry

## Abstract

This article comments on:

**Kim J, Yang R, Chang C, Park Y, Tucker ML.** 2018. The root-knot nematode (*Meloidogyne incognita*) produces a functional mimic of the Arabidopsis IDA signaling peptide. Journal of Experimental Botany 69, 3009–3021.

**Plant pathogens have evolved diverse strategies to manipulate and co-opt host cellular function to enable infection. One strategy commonly employed by plant-parasitic cyst and root-knot nematodes is molecular mimicry of host proteins and small-molecule ligands. In an important new example of this phenomenon**, **Kim *et al.* (2018)****have now identified a putatively secreted peptide from the root-knot nematode *Meloidogyne incognita* that mimics the Arabidopsis INFLORESCENCE DEFICIENT IN ABSCISSION (IDA) signaling peptide, which controls floral organ abscission and lateral root emergence.**

Phytopathogens have evolved sophisticated mechanisms to enhance their ability to infect host plants. These include the molecular mimicry of cellular activities and functions, the ability to encode factors that mirror host proteins in function or structure to manipulate cellular functions for the pathogen’s benefit. Such mimicry may enable the pathogen to establish infection while escaping the host’s immune detection system. Although molecular mimicry is a common strategy employed by bacterial and viral pathogens in various animal systems (Stebbins and Galán, 2001; Elde and Malik, 2009), information about this phenomenon in phytopathogens is relatively limited.

The plant-parasitic cyst and root-knot nematodes, unlike other plant pathogens, appear to rely heavily on molecular mimicry to parasitize host plants (Hewezi, 2015). An example of cyst nematode mimicry is the secretion of effector proteins containing a conserved 12-amino acid C-terminal motif sharing strong sequence similarity with plant CLAVATA3/ESR (CLE) ligand peptides (Mitchum *et al.*, 2012). Similarly, multiple tandemly arrayed CLE-like motifs have been identified in various members of the secreted *Meloidogyne Avirulence Protein* (MAP) family (Rutter *et al.*, 2014). Thus, plant CLE ligands represent weak common targets for mimicry by two evolutionarily diverse species of plant-parasitic nematodes.

Other widely distributed mimics produced by plant-parasitic nematodes are the secreted chorismate mutases (Doyle and Lambert, 2003; Huang *et al.*, 2005; Vanholme *et al.*, 2009). Because of the absence of its substrate in animals, it is anticipated that parasitic nematodes mimic host chorismate mutase to alter secondary metabolic pathways, presumably to interfere with defense-related functions. Another effector that may mimic a host protein to subvert the defense response is the annexin-like effector from cyst nematodes (Patel *et al.*, 2010). It can complement the Arabidopsis *annexin1* mutant despite low amino acid sequence identity between the two proteins (Patel *et al.*, 2010). The identification of a number of effectors with putative mimicry functions from plant-parasitic nematodes suggests that these parasites use molecular mimicry to interfere with and/or exploit essential cellular functions required for parasitism.

## The origin of nematode-encoded mimics

Horizontal gene transfer (HGT) seems to be the most plausible source for nematode mimics, with several potential such events having been reported in plant-parasitic nematodes (Scholl *et al.*, 2003, Danchin *et al.*, 2010; Haegeman *et al.*, 2011). This type of mimicry can be detected through sequence similarity and phylogeny. However, it is reasonable to expect that in some cases, because of the rapid evolution of parasitic nematodes, the nematode-encoded mimics may have little or no similarity to host factors that they imitate. Also, in nematode-encoded mimics generated through convergent evolution, parasitic nematodes independently evolve to acquire functions or structures that mirror those of host plants with limited sequence similarity.

Plant-parasitic nematodes can also use structural mimicry to co-opt host post-translational modification mechanisms to the nematode’s advantage. One example is the cyst nematode effector 10A07, which appears to structurally mimic a plant kinase substrate in order to become phosphorylated inside plant cells (Hewezi *et al.*, 2015). Phosphorylation of the 10A07 effector is required for its translocation and function in the nucleus. Since pathogen-encoded structural mimics have little or no sequence similarity to the host factors that they model, their identification remains challenging and mainly relies on functional and structural analyses.

## *Meloidogyne incognita* IDA-like peptide and mimicry of INFLORESCENCE DEFICIENT IN ABSCISSION

Kim *et al.* (2018) provide another intriguing example of how root-knot nematodes may hijack signaling pathways to promote parasitism. Two putatively secreted peptides with sequence similarity to Arabidopsis INFLORESCENCE DEFICIENT IN ABSCISSION (IDA) have been identified from the root-knot nematode *Meloidogyne incognita* (Kim *et al.*, 2018). The *M. incognita IDA-like* genes, *MiIDL1* and *MiIDL2*, encode a small protein with N-terminal signal peptide for secretion. Several studies have demonstrated the function of the IDA signaling peptide in cell separation that mediates floral organ abscission and lateral root emergence (Butenko *et al.*, 2003; Cho *et al.*, 2008; Santiago *et al.*, 2016), presumably through the regulation of various cell well-modifying enzymes (Kumpf *et al.*, 2013). The IDA peptide functions as a ligand of two functionally redundant plasma membrane-localized receptor-like kinases known as HAE and HSL2. IDA binding to the receptors initiates a signaling pathway leading to gene expression changes through the activity of a set of KNOX transcription factors (Liljegren, 2012; Niederhuth *et al.*, 2013; Meng *et al.*, 2016).

Similar to its Arabidopsis counterpart, MiIDL1 is assumed to be processed to a 22-amino acid bioactive peptide. Interestingly, exogenous application of synthetic MiIDL1 peptide meaningfully rescued the abscission phenotype of the Arabidopsis *ida* mutant, providing the first indication of a mimicry function of MiIDL1 in host cells. In addition, genetic complementation of the Arabidopsis *ida* mutant with the full-length *MiIDL1* gene containing the N-terminal signal peptide sequence produced the wild-type floral organ abscission phenotype (Kim *et al.*, 2018). The importance of MiIDL1 for nematode pathogenesis was demonstrated by data showing that silencing *MiIDL1* transcripts in the nematode through a host-induced RNAi approach significantly reduced the number and size of nematode-induced galls. It seems most likely that during nematode infection MiIDL1 peptide mimics IDA function in particular root cell types that are not usually subjected to an IDA-mediated signaling pathway. However, it remains to be determined whether MiIDL1 peptide exploits host HAE/HSL2 receptors during infection or utilizes different receptors with similar functions, taking into consideration the low sequence similarity between IDA and MiIDL1.

## The power of being imperfect

The sequence identity between MiIDL1 and the mimicked plant IDA is low and limited to the conserved C-terminal motif. This situation is characteristic of convergent evolution, although the possibility that the mimicry function of MiIDL1 was acquired via divergent evolution cannot be ruled out. Regardless of evolutionary origin, the ability of *MiIDL1* to complement the Arabidopsis *ida* mutant, despite strong sequence divergence, is fascinating. The low sequence similarity between MiIDL1 and IDA may enable the nematode to mirror the function of IDA sufficiently without falling completely under the control of host cellular regulatory mechanisms.

In this context, pathogen mimics that show perfect or near-perfect sequence similarity to host factors (such as cyst nematode CLE-like peptides) may be speculated to be less advantageous to the pathogens because of their limited ability to disrupt cellular activity to the benefit of the pathogen while falling under host cellular control. Conversely, being too divergent may enable the plant immune system to identify pathogen mimics. Thus, co-opting or subverting host cellular functions to the pathogen’s benefit without being recognized appears to be the most advantageous tactic for the pathogen, though this has yet to be determined experimentally.

## New targets for generating nematode resistance

IDA genes are highly conserved in plants and the encoded peptides may represent vulnerable hotspots for the nematode to hijack IDA-mediated signaling pathways and establish infection. Highly conserved genes neverthless often undergo a small amount of ‘evolutionary flexibility’ and this may limit the plant’s ability to escape mimicry. Nevertheless, even with this level of sequence variation, the host plants may acquire substitutions in certain IDA-peptide residues that enable them to evade mimicry without affecting their binding affinity to HAE or HSL2. In this context, complementation of the Arabidopsis *ida1* mutant with IDA1 transgenes containing point mutations in various conserved amino acids may lead to the identification of residues that enable the plants to evade mimicry without compromising the interaction ability of IDA with HAE or HSL2. These conserved residues would provide excellent targets for generating nematode resistance through non-GMO genome editing approaches.

The use of mimicry by parasitic nematodes to recognize and gain entry to host plants is intriguing and well worth additional exploration since interference of host–pathogen recognition may lead to innovative nematode control strategies. With the rapid development of sequencing technologies the genome sequences of a significant number of phytopathogens and their host plants have now become available, and so it is possible to explore the function of molecular mimicry in other plant pathosystems. This should lead to the identification of attractive targets for disease resistance.
